# Associations of Subcutaneous and Visceral Fat Areas with Progression-Free Survival in Patients with Diffuse Large B-Cell Lymphoma

**DOI:** 10.7150/ijms.137961

**Published:** 2026-09-24

**Authors:** Jiun-Hung Geng, Chien-Hsiang Chang, Yi-Chang Liu, Hui-Hua Hsiao, Tsung-Jang Yeh, Jeng-Shiun Du, Min-Hong Wang, Yu-Yin Lin, Wen-Hsin Chang, Chin-Mu Hsu, Chun-Hung Richard Lin, Shih-Feng Cho

**Affiliations:** 1 Department of Urology, Kaohsiung Municipal Siaogang Hospital, Kaohsiung 812, Taiwan.; 2 Department of Urology, Kaohsiung Medical University Hospital, Kaohsiung Medical University, Kaohsiung 807, Taiwan.; 3 Center for Big Data Research, Kaohsiung Medical University 807, Kaohsiung, Taiwan.; 4 Department of Computer Science and Engineering, National Sun Yat-Sen University, Kaohsiung 804, Taiwan.; 5 Division of Hematology and Oncology, Department of Internal Medicine, Kaohsiung Medical University Hospital, Kaohsiung Medical University, Kaohsiung 807, Taiwan.; 6 Department of Occupational Safety and Health, Kaohsiung Medical University Hospital, Kaohsiung Medical University, Kaohsiung 807, Taiwan.; 7 Faculty of Medicine, College of Medicine, Kaohsiung Medical University, Kaohsiung 807, Taiwan.; 8 Center for Cancer Research, Kaohsiung Medical University, Kaohsiung 807, Taiwan.

**Keywords:** diffuse large B-cell lymphoma, subcutaneous fat area, visceral fat area, body composition, progression-free survival

## Abstract

**Background:**

Body composition, particularly adipose tissue distribution, may modulate cancer progression and therapeutic outcomes. This study investigated the associations of subcutaneous fat area (SFA) and visceral fat area (VFA) with progression-free survival (PFS) in patients with diffuse large B-cell lymphoma (DLBCL).

**Methods:**

This retrospective study included 110 patients with DLBCL diagnosed between 2010 and 2019. SFA and VFA were quantified from baseline computed tomography images using a previously validated automated deep learning-based segmentation system and analyzed as continuous variables standardized to 1-standard deviation (SD) increments. Associations with PFS were evaluated using Cox proportional hazards regression adjusted for the International Prognostic Index (IPI), with joint modeling, sensitivity analyses, restricted cubic spline analysis, and bootstrap internal validation. Overall survival (OS) was additionally evaluated in exploratory analyses.

**Results:**

During a mean follow-up of 63.5 ± 45.2 months, 46 PFS events (41.8%) were observed. In separate IPI-adjusted models, each 1-SD increase in SFA was associated with poorer PFS (hazard ratio [HR], 1.34; 95% confidence interval [CI], 1.00-1.79; p = 0.047), whereas VFA was not significantly associated with PFS (HR, 1.09; 95% CI, 0.82-1.44; p = 0.552). In the joint model, the SFA estimate remained similar (HR, 1.33; 95% CI, 0.99-1.79; p = 0.057), whereas the VFA estimate remained close to the null (HR, 1.03; 95% CI, 0.76-1.38; p = 0.868); the standardized coefficients did not differ significantly (p = 0.265). Adding SFA to the IPI yielded a small increase in the apparent C-index (0.618 to 0.632; ΔC-index, 0.014; bootstrap 95% CI, -0.013 to 0.069). In exploratory analyses, neither SFA nor VFA was significantly associated with OS.

**Conclusion:**

Higher SFA was associated with poorer PFS after adjustment for the IPI, whereas VFA showed little evidence of an association; however, the difference between their associations was not statistically significant, and neither adipose tissue compartment was significantly associated with OS. The incremental prognostic value of SFA beyond the IPI was limited. Larger studies with external validation are needed to confirm these findings.

## Introduction

Diffuse large B-cell lymphoma (DLBCL), accounting for approximately 30-40% of non-Hodgkin lymphomas, is one of the most common hematologic malignancies worldwide and remains a major cause of cancer-related morbidity and mortality 1, 2. The development of immunochemotherapy, especially regimens that incorporate the anti-CD20 antibody rituximab (R) into conventional chemotherapy, such as R-CHOP (rituximab, cyclophosphamide, doxorubicin, vincristine, and prednisone), has substantially improved treatment response and patient outcomes and is considered one of the main standards for first-line treatment 2-4. However, treatment responses remain highly heterogeneous due to the complex pathogenesis and genetic heterogeneity, with some patients achieving durable remission while others experience early disease progression.

Risk stratification and treatment decision-making in DLBCL continue to evolve with advances in diagnostic technologies. Currently, the International Prognostic Index (IPI) 5, which incorporates age, serum lactate dehydrogenase (LDH), ECOG performance status, Ann Arbor stage, and number of extranodal sites, remains the most widely used clinical tool for predicting outcomes and stratifying patients in clinical trials. Refinements such as the NCCN-IPI 6, which introduces weighted scoring for age, LDH ratio, and specific extranodal sites, have demonstrated superior discrimination between risk groups. In addition, advances in molecular profiling have revealed that DLBCL encompasses distinct biologic entities with divergent outcomes. Cell-of-origin classification by gene expression profiling distinguishes the germinal center B-cell subtype from the activated B-cell subtype, with the latter associated with a poorer prognosis 7, 8. Moreover, the development of next-generation sequencing and bioinformatics enables integrative genomic analyses in large patient cohorts to identify prognostically relevant genetic alterations and establish novel genetic classifications 9-11. Recently, emerging tools, such as PET-derived metabolic tumor volume and circulating tumor DNA dynamics, have shown promise in outperforming clinical indices for risk prediction 12. However, these novel molecular and imaging-based biomarkers have yet to be integrated into a validated prognostic index, and their clinical implementation remains limited by restricted accessibility and high technical requirements. Therefore, identifying additional predictors that can improve risk stratification remains an important and unmet clinical need.

In recent years, increasing attention has been directed toward the role of body composition in cancer prognosis, treatment response, and clinical outcomes 13-15. Beyond traditional measures such as body mass index (BMI), specific fat distributions, including visceral fat area (VFA) and subcutaneous fat area (SFA), are more closely associated with metabolic dysregulation, systemic inflammation, and tumor progression 16-18. Emerging evidence suggests that adipose tissue is closely related not only to cancer development but also to the efficacy of anticancer therapies, potentially through alterations in the tumor microenvironment, drug pharmacokinetics, and immune regulation 19-22. Of note, visceral and subcutaneous fat depots may have distinct biological effects, and accumulating evidence suggests that fat distribution may provide prognostic information not captured by BMI 23-25. While several studies have linked the distribution of visceral adiposity or subcutaneous fat to clinical outcomes in solid tumors 26-30, the prognostic significance of these parameters in hematologic malignancies, especially in lymphoma, remains to be elucidated.

Previous studies have indicated that body-composition characteristics, including adipose tissue distribution and skeletal muscle mass, are associated with clinical outcomes in DLBCL 31, 32. For example, factors such as a low body mass index (BMI <18.5 kg/m²), a high visceral adipose tissue ratio, and the presence of sarcopenia have been associated with inferior survival 33, 34. However, the prognostic significance of distinct adipose tissue compartments remains incompletely understood. We therefore hypothesized that SFA and VFA may be associated with clinical outcomes in DLBCL. To test this hypothesis, we quantified SFA and VFA from computed tomography (CT) images using an automated deep learning-based segmentation system and investigated their associations with PFS. We further evaluated whether SFA provided incremental prognostic information beyond the IPI, while overall survival (OS) was additionally examined as an exploratory outcome.

## Patients and Methods

### Study cohort

Patients diagnosed with DLBCL were retrospectively identified from Kaohsiung Medical University Hospital between April 2010 and October 2019. A total of 110 patients were included in the study. Clinical data, including demographic characteristics, laboratory findings, treatment regimens, and disease progression status, were collected from electronic medical records. In addition, CT images were retrieved for body composition analysis. All patients underwent standard clinical follow-up after diagnosis and treatment. This study was approved by the Institutional Review Board of Kaohsiung Medical University Hospital (KMUHIRB-E(I)-20220021).

### Ct-based body composition assessment

During the study period, abdominal CT examinations were acquired using a standardized spiral CT protocol with 5-mm collimation and 2.5-mm reconstruction. For body composition analysis, a single axial image at the level of the umbilicus was selected for quantification of abdominal adiposity. The umbilical level was chosen based on established CT-based approaches for quantifying visceral and subcutaneous adipose tissue, including previous studies in patients with cancer 35-38. Although anatomical landmarks for adipose tissue assessment vary across studies, several lumbar and abdominal levels have been used for single-slice adiposity assessment 35-38. VFA and SFA were quantified using a previously developed and validated automated CT image-analysis system based on a TransUNet deep-learning architecture 39, 40. In the original validation study, the segmentation model was trained using 2,020 manually annotated abdominal CT images and achieved Dice coefficients of 0.956 and 0.984 for visceral and subcutaneous adipose tissue, respectively, with corresponding mean intersection-over-union values of 0.952 and 0.982 39, 40. The previously validated segmentation framework was applied without modification in the present study and performed automated pixel-wise segmentation of the visceral and subcutaneous adipose tissue compartments, from which the corresponding fat areas were calculated using standardized pixel-to-area scaling. All generated segmentation maps were visually inspected as a quality-control procedure to identify obvious segmentation errors or image-quality issues. Representative segmentation outputs and patterns of fat distribution are shown in Supplementary Figure S1 (A-C). SFA and VFA were treated as continuous variables in the survival analyses. To facilitate comparison of effect estimates between the two adipose tissue compartments, both measures were standardized, and hazard ratios (HRs) were expressed per 1-standard deviation (SD) increase.

### Clinical outcome

The primary outcome of this study was PFS. PFS was defined as the time from the date of diagnosis to the first documented disease progression, relapse, or death from any cause, whichever occurred first. Disease progression or relapse was determined based on clinical evaluation, imaging findings, and/or pathological confirmation, in accordance with standard response assessment criteria for lymphoma. Patients without a PFS event were censored at the date of last follow-up.

### Covariates and variable definitions

Clinical variables considered in the study included demographic factors (age and sex), nutritional and metabolic status (BMI), performance status (ECOG), disease burden (Ann Arbor stage), comorbidity status (Charlson Comorbidity Index [CCI]), treatment regimen (R-CHOP vs non-R-CHOP), and laboratory parameters reflecting tumor activity and organ function, including lactate dehydrogenase (LDH) and serum creatinine. The “non-R-CHOP” regimens include other less intensive treatments, such as R-COP or rituximab-based combination treatment. Body composition parameters, including VFA and SFA, were included as key variables of interest given their potential roles in cancer metabolism and treatment response. The IPI was used as the principal clinical prognostic measure in the primary survival models because it integrates established lymphoma-related prognostic information while allowing parsimonious model specification given the number of observed events.

### Statistical analysis

Continuous variables are presented as mean ± SD and were compared using the independent t-test, while categorical variables are expressed as frequencies (percentages) and were compared using the chi-square test.

Associations of SFA and VFA with PFS were evaluated using Cox proportional hazards regression models. To minimize model overfitting and facilitate direct comparison between adipose tissue compartments, SFA and VFA were analyzed as continuous variables standardized to 1-SD increments. Separate parsimonious models were first fitted for SFA and VFA, with each model adjusted for the IPI. Analyses were conducted using a common complete-case cohort with available PFS, IPI, SFA, and VFA data.

A joint fat-compartment model including both standardized SFA and VFA together with the IPI was subsequently fitted to evaluate their associations when mutually adjusted. The difference between the standardized SFA and VFA regression coefficients was formally evaluated using a Wald test of the null hypothesis that the two coefficients were equal.

Because SFA showed the stronger estimated association with PFS in the primary analyses, additional analyses focused on characterizing the SFA association. The proportional hazards assumption for the primary IPI-plus-SFA model was evaluated using Schoenfeld residuals. Potential nonlinearity in the association between SFA and PFS was examined using a restricted cubic spline with three knots, adjusted for the IPI, with the median SFA value used as the reference. Overall and nonlinear associations were evaluated using Wald tests.

Two sensitivity models were used to examine the robustness of the SFA association. A clinical sensitivity model additionally included CCI and treatment regimen together with the IPI, whereas a body-size and adiposity sensitivity model additionally included VFA and BMI. Correlations among SFA, VFA, and BMI were assessed using Pearson correlation coefficients, and variance inflation factors were examined to assess potential multicollinearity.

To evaluate whether SFA provided incremental prognostic information beyond the IPI, the IPI-only model was compared with the IPI-plus-SFA model using Harrell's C-index, the Akaike information criterion (AIC), and a likelihood-ratio test. The change in C-index was calculated, with its 95% confidence interval estimated using 1,000 bootstrap resamples. Internal validation was performed using 1,000 bootstrap resamples to estimate optimism-corrected C-indices. Calibration of the IPI-plus-SFA model for 3-year PFS was assessed using bootstrap resampling with 1,000 repetitions.

As an exploratory secondary analysis, associations of SFA and VFA with OS were evaluated using the same Cox proportional hazards modeling framework as the primary PFS analysis. OS was defined as the time from diagnosis to death from any cause, with patients alive at the last follow-up censored at that date. Separate IPI-adjusted models were fitted for standardized SFA and VFA, followed by a joint model including the IPI and both adipose tissue compartments; their standardized coefficients were compared using the same Wald test described above. The proportional hazards assumption was evaluated using Schoenfeld residuals.

All statistical tests were two-sided, and p < 0.05 was considered statistically significant. Analyses were performed using R version 4.4.2 (R Foundation for Statistical Computing, Vienna, Austria).

## Results

### Baseline characteristics

A total of 110 patients with DLBCL were included in this analysis. During the follow-up period, 46 PFS events (41.8%) were observed, while 64 patients (58.2%) remained progression-free during a mean follow-up of 63.5 ± 45.2 months. Baseline characteristics according to PFS event status are summarized in Table 1. Compared with patients without a PFS event, those who experienced a PFS event more frequently had advanced-stage disease (stage III-IV: 73.9% vs 54.7%, p = 0.040), a higher comorbidity burden (CCI ≥2: 60.9% vs 37.5%, p = 0.015), and received non-R-CHOP regimens more frequently (39.1% vs 15.6%, p = 0.003). Serum creatinine levels were also higher in patients with a PFS event (1.09 ± 0.68 vs 0.89 ± 0.29 mg/dL, p = 0.041). No statistically significant differences were observed in age, BMI, sex, ECOG performance status, LDH, VFA, or SFA between the two groups.

### Associations of subcutaneous and visceral fat areas with progression-free survival

Among the 109 patients with complete data for the primary survival analysis, 46 PFS events were observed. SFA and VFA were analyzed as continuous variables standardized to 1-SD increments. In separate Cox proportional hazards models adjusted for the IPI, each 1-SD increase in SFA was associated with a higher hazard of a PFS event (HR, 1.34; 95% CI, 1.00-1.79; p = 0.047), whereas VFA was not significantly associated with PFS (HR, 1.09; 95% CI, 0.82-1.44; p = 0.552) (Table 2). In the joint model including both adipose tissue compartments and the IPI, the magnitude of the association for SFA remained similar (HR, 1.33; 95% CI, 0.99-1.79; p = 0.057), whereas the estimate for VFA remained close to the null (HR, 1.03; 95% CI, 0.76-1.38; p = 0.868). However, a formal Wald test showed no statistically significant difference between the standardized SFA and VFA coefficients (p = 0.265).

### Functional form and proportional hazards assumption for subcutaneous fat area

There was no evidence of violation of the proportional hazards assumption for the primary IPI-adjusted SFA model, either for IPI (p = 0.750), SFA (p = 0.900), or globally (p = 0.950). Restricted cubic spline analysis with three knots showed no evidence of a nonlinear association between SFA and PFS (p for nonlinearity = 0.978; p for overall association = 0.141), with the median SFA value of 153 cm² used as the reference (Figure 1).

### Sensitivity analyses

The association between SFA and PFS was further examined in sensitivity analyses (Supplementary Table S1). In the clinical sensitivity model additionally accounting for CCI and treatment regimen, each 1-SD increase in SFA was associated with a higher hazard of a PFS event (HR, 1.52; 95% CI, 1.11-2.08; p = 0.009). In the body-size and adiposity sensitivity model additionally including VFA and BMI, the association was attenuated (HR, 1.22; 95% CI, 0.87-1.70; p = 0.250). Correlations among the adiposity measures were modest to moderate, with Pearson correlation coefficients of 0.223 between SFA and VFA, 0.531 between SFA and BMI, and 0.426 between VFA and BMI. Variance inflation factors ranged from 1.05 to 1.65, indicating no substantial multicollinearity in the body-size and adiposity sensitivity model.

### Incremental prognostic value and internal validation

The incremental prognostic value of SFA beyond the IPI was evaluated by comparing the IPI-only model with the IPI-plus-SFA model (Table 3). The apparent Harrell's C-index increased from 0.618 for the IPI-only model to 0.632 after the addition of SFA, corresponding to a ΔC-index of 0.014 (bootstrap 95% CI, -0.013 to 0.069). The AIC decreased from 402.2 to 400.5, while the likelihood-ratio test comparing the two nested models yielded a p-value of 0.053. After 1,000 bootstrap resamples, the optimism-corrected C-index was 0.617 for the IPI-only model and 0.620 for the IPI-plus-SFA model. Bootstrap-corrected calibration of the IPI-plus-SFA model for 3-year PFS is shown in Supplementary Figure S2.

### Exploratory overall survival analysis

During follow-up, 43 deaths were observed. In exploratory analyses, neither SFA nor VFA was significantly associated with OS in the IPI-adjusted models (SFA: HR per 1-SD increase, 1.13; 95% CI, 0.82-1.54; p = 0.459; VFA: HR, 1.07; 95% CI, 0.79-1.43; p = 0.678) or in the joint model (SFA: HR, 1.12; 95% CI, 0.81-1.54; p = 0.507; VFA: HR, 1.04; 95% CI, 0.76-1.42; p = 0.799) (Supplementary Table S2). The standardized SFA and VFA coefficients did not differ significantly (p = 0.784), with no evidence of violation of the proportional hazards assumption (global p ≥ 0.86).

## Discussion

In this study, higher SFA was associated with poorer PFS in patients with DLBCL after adjustment for the IPI, whereas VFA showed little evidence of an association under the same modeling framework. When SFA and VFA were included simultaneously, the SFA effect estimate remained largely unchanged, although its confidence interval included the null, while the VFA estimate remained close to unity. However, the standardized SFA and VFA coefficients did not differ significantly, precluding a definitive conclusion that their associations with PFS differed between adipose tissue compartments. Moreover, adding SFA to the IPI provided only limited incremental prognostic information. In exploratory analyses, however, neither SFA nor VFA was significantly associated with OS, indicating that the association observed for SFA with PFS was not reproduced for all-cause mortality. These findings suggest that SFA may be associated with DLBCL prognosis, but its prognostic contribution beyond established clinical information requires further validation.

SFA showed a stronger estimated association with PFS than VFA, although the difference between their standardized coefficients was not statistically significant. This pattern may nevertheless raise questions regarding whether different adipose tissue compartments have distinct biological relevance in DLBCL. Adipose tissue is an active endocrine and metabolic organ that can influence systemic inflammation, immune regulation, and cancer-related signaling 41-45. In particular, subcutaneous adipose tissue is an important source of circulating leptin, which has been implicated in PI3K/AKT signaling and the regulation of Bcl-2 and cyclin D1 in malignant B cells 46, 47. Body composition may also influence the pharmacokinetics and distribution of anticancer therapies, including rituximab and cytotoxic agents 48-52. These mechanisms provide biological plausibility for an association between adipose distribution and DLBCL outcomes. However, they remain speculative in the context of the present study, in which circulating adipokines, inflammatory markers, and treatment pharmacokinetics were not directly measured.

In addition to body composition, several established clinical characteristics were associated with PFS event status in our cohort. Patients who experienced a PFS event more frequently had advanced-stage disease and greater comorbidity burden and were more likely to receive non-R-CHOP regimens. These findings are broadly consistent with previous studies identifying disease burden and comorbidity as important determinants of outcomes in DLBCL 5, 6, 53, 54. In the present analysis, the association between SFA and PFS remained evident after adjustment for the IPI and in the clinical sensitivity model additionally accounting for CCI and treatment regimen. The persistence of the SFA association across these models suggests that it was not fully accounted for by the clinical prognostic factors included in these analyses.

Alternatively, SFA may represent a broader host metabolic phenotype rather than a direct biological driver of lymphoma progression. Subcutaneous adiposity is related to systemic metabolic and inflammatory alterations and may capture aspects of host physiology that are not fully represented by conventional anthropometric measures such as BMI. The attenuation of the SFA association after additional adjustment for BMI and VFA in our study is consistent with at least partial overlap among these measures of body size and adiposity. Therefore, SFA should not necessarily be interpreted as a causal factor, but rather as a potential imaging-derived marker of host characteristics relevant to DLBCL outcomes.

From a prognostic modeling perspective, the addition of SFA to the IPI provided only limited incremental information. Although the apparent C-index increased from 0.618 to 0.632 and the AIC decreased modestly, the bootstrap confidence interval for the change in C-index included zero, and the likelihood-ratio test did not reach conventional statistical significance. Moreover, after bootstrap correction for optimism, the difference in C-index between the IPI-only and IPI-plus-SFA models was small. These findings indicate that the observed association between SFA and PFS does not necessarily translate into meaningful improvement in prognostic performance beyond the IPI alone 55. Further evaluation in larger, independent cohorts is needed to determine whether CT-derived SFA can contribute to prognostic assessment in DLBCL 56.

Several limitations of this study should be acknowledged. First, this was a retrospective, single-center study with a modest sample size (110 patients) and a limited number of PFS events (n = 46) and deaths (n = 43). This may have limited statistical power to detect modest differences between the associations of SFA and VFA, small incremental improvements in prognostic discrimination, and more modest associations with OS, while also limiting the generalizability of our findings. Second, although the primary models were adjusted for the IPI and additional clinical and body composition-related factors were considered in sensitivity analyses, residual and unmeasured confounding cannot be excluded. Skeletal muscle mass and muscle radiation attenuation were not quantified in this cohort; therefore, potential confounding by sarcopenia, myosteatosis, or sarcopenic obesity could not be evaluated. Third, body composition was assessed using a single CT slice at the umbilical level. Although the umbilical and lower lumbar levels have been used in previous CT-based assessments of abdominal adiposity, anatomical landmarks for adipose tissue quantification vary across studies; therefore, differences in slice selection may limit direct comparability with studies using other anatomical levels. Moreover, single-slice measurements may not fully capture whole-body adiposity. In patients with bulky retroperitoneal or pelvic lymphoma, local anatomical distortion or displacement of adipose tissue could introduce measurement variability, although all segmentation outputs underwent visual quality control. Dynamic changes in body composition during treatment were also not evaluated. Fourth, the study cohort was treated between 2010 and 2019, and evolving therapeutic strategies for DLBCL may limit the applicability of our findings to contemporary treatment settings. Finally, although bootstrap resampling was used for internal validation, an independent external validation cohort was not available, and the incremental prognostic value of SFA beyond the IPI was limited. Nevertheless, SFA can be derived from routinely acquired CT images without additional imaging procedures or patient burden, supporting its feasibility as an imaging-derived measure for further investigation. Larger, prospective, multicenter studies incorporating comprehensive assessment of both adipose and skeletal muscle compartments are needed to confirm these findings and determine whether CT-derived body-composition measures provide clinically useful prognostic information beyond established risk factors.

## Conclusions

In conclusion, higher SFA was associated with poorer PFS in patients with DLBCL after adjustment for the IPI, whereas VFA showed little evidence of an association. However, the difference between the standardized SFA and VFA associations was not statistically significant, and the incremental prognostic value of SFA beyond the IPI was limited. Larger, prospective studies with external validation are needed to confirm these findings and determine the biological and clinical relevance of CT-derived adiposity measures in DLBCL.

## Supplementary Material

Supplementary figures and tables.

## Figures and Tables

**Figure 1:**
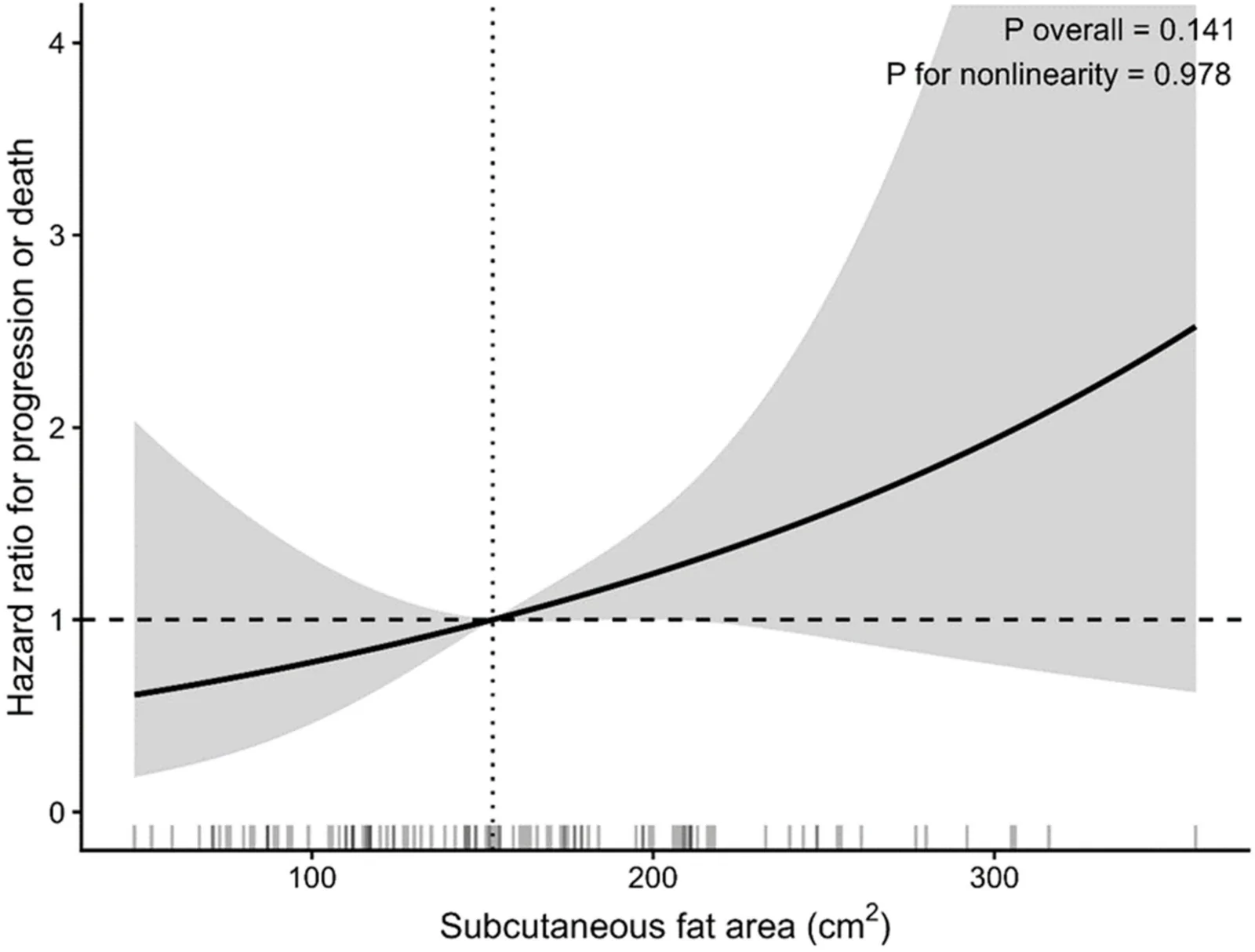
** Restricted Cubic Spline Analysis of the Association Between Subcutaneous Fat Area and Progression-Free Survival.** Restricted cubic spline analysis of the association between subcutaneous fat area (SFA) and progression-free survival, adjusted for the International Prognostic Index (IPI). The spline was modeled using three knots, with the median SFA value (153 cm²) as the reference (hazard ratio [HR] = 1.0). The solid line represents the estimated hazard ratio and the shaded area represents the 95% confidence interval. There was no evidence of nonlinearity (p for nonlinearity = 0.978; p for overall association = 0.141).

**Table 1:** Baseline Characteristics According to Progression-Free Survival Event Status

Variable	All patients (n = 110)	No PFS Event (n = 64)	PFS Event (n = 46)	*p* value
Age (years)	60.50 ± 14.02	58.73 ± 13.86	62.96 ± 14.02	0.120
BMI (kg/m²)	24.35 ± 3.53	23.85 ± 3.38	25.05 ± 3.66	0.078
Sex				0.129
Female	60	31 (48.4%)	29 (63.0%)	
Male	50	33 (51.6%)	17 (37.0%)	
ECOG status				0.058
0-1	100	61 (95.3%)	39 (84.8%)	
≥ 2	10	3 (4.7%)	7 (15.2%)	
Ann Arbor stage				0.040
I-II	41	29 (45.3%)	12 (26.1%)	
III-IV	69	35 (54.7%)	34 (73.9%)	
CCI				0.015
0-1	58	40 (62.5%)	18 (39.1%)	
≥2	52	24 (37.5%)	28 (60.9%)	
Treatment regimen				0.003
R-CHOP	82	54 (84.4%)	28 (60.9%)	
non-R-CHOP	28	10 (15.6%)	18 (39.1%)	
LDH (U/L)	296.89 ± 194.92	311.81 ± 225.94	276.13 ± 140.74	0.311
Creatinine (mg/dL)	0.97 ± 0.50	0.89 ± 0.29	1.09 ± 0.68	0.041
VFA (cm^2^)	111.76 ± 50.06	108.25 ± 53.87	116.65 ± 44.33	0.388
SFA (cm^2^)	160.87 ± 64.12	152.89 ± 62.55	171.98 ± 65.29	0.124

Abbreviations: PFS, progression-free survival; BMI, body mass index; ECOG, Eastern Cooperative Oncology Group performance status; CCI, Charlson Comorbidity Index; LDH, lactate dehydrogenase; VFA, visceral fat area; SFA, subcutaneous fat area; R-CHOP, rituximab, cyclophosphamide, doxorubicin, vincristine, and prednisone. Note: PFS events included disease progression, relapse, or death from any cause, whichever occurred first. Continuous variables are presented as mean ± standard deviation. Categorical variables are presented as n (column %), with percentages calculated within each PFS event-status group.

**Table 2:** Associations of Subcutaneous and Visceral Fat Areas with Progression-Free Survival

Model	Variable	HR per 1-SD increase	95% CI	*p* value
Separate IPI-adjusted models				
Model 1	SFA	1.34	1.00-1.79	0.047
Model 2	VFA	1.09	0.82-1.44	0.552
Joint fat-compartment model				
Model 3	SFA	1.33	0.99-1.79	0.057
	VFA	1.03	0.76-1.38	0.868

Abbreviations: HR, hazard ratio; CI, confidence interval; SD, standard deviation; SFA, subcutaneous fat area; VFA, visceral fat area; IPI, International Prognostic Index. Note: Analyses were performed in 109 patients with complete data for IPI, SFA, and VFA, with 46 PFS events. SFA and VFA were analyzed as continuous variables standardized to 1-SD increments. Models 1 and 2 included IPI and either SFA or VFA, respectively. Model 3 included IPI, SFA, and VFA simultaneously. The standardized SFA and VFA regression coefficients in the joint model did not differ significantly (Wald test, p = 0.265).

**Table 3:** Incremental Prognostic Value of SFA Beyond the International Prognostic Index

Model	Apparent C-index	Optimism-corrected C-index	AIC	ΔC-index (95% CI)	LRT *p* value
IPI only	0.618	0.617	402.2	-	-
IPI + SFA	0.632	0.620	400.5	0.014 (-0.013 to 0.069)	0.053

Abbreviations: SFA, subcutaneous fat area; IPI, International Prognostic Index; AIC, Akaike information criterion; LRT, likelihood-ratio test; CI, confidence interval. Note: The ΔC-index represents the difference in apparent Harrell's C-index between the IPI-plus-SFA and IPI-only models; its 95% confidence interval was estimated using 1,000 bootstrap resamples. Optimism-corrected C-indices were estimated using 1,000 bootstrap resamples. The LRT compares the nested IPI-only and IPI-plus-SFA models.

## References

[1] Sehn LH, Salles G (2021). Diffuse Large B-Cell Lymphoma. N Engl J Med.

[2] Silkenstedt E, Salles G, Campo E, Dreyling M (2024). B-cell non-Hodgkin lymphomas. Lancet.

[3] Coiffier B, Lepage E, Briere J, Herbrecht R, Tilly H, Bouabdallah R (2002). CHOP chemotherapy plus rituximab compared with CHOP alone in elderly patients with diffuse large-B-cell lymphoma. N Engl J Med.

[4] Pfreundschuh M, Kuhnt E, Trumper L, Osterborg A, Trneny M, Shepherd L (2011). CHOP-like chemotherapy with or without rituximab in young patients with good-prognosis diffuse large-B-cell lymphoma: 6-year results of an open-label randomised study of the MabThera International Trial (MInT) Group. Lancet Oncol.

[5] International Non-Hodgkin's Lymphoma Prognostic Factors P (1993). A predictive model for aggressive non-Hodgkin's lymphoma. N Engl J Med.

[6] Ruppert AS, Dixon JG, Salles G, Wall A, Cunningham D, Poeschel V (2020). International prognostic indices in diffuse large B-cell lymphoma: a comparison of IPI, R-IPI, and NCCN-IPI. Blood.

[7] Rosenwald A, Wright G, Chan WC, Connors JM, Campo E, Fisher RI (2002). The use of molecular profiling to predict survival after chemotherapy for diffuse large-B-cell lymphoma. N Engl J Med.

[8] Lenz G, Wright G, Dave SS, Xiao W, Powell J, Zhao H (2008). Stromal gene signatures in large-B-cell lymphomas. N Engl J Med.

[9] Chapuy B, Stewart C, Dunford AJ, Kim J, Kamburov A, Redd RA (2018). Molecular subtypes of diffuse large B cell lymphoma are associated with distinct pathogenic mechanisms and outcomes. Nat Med.

[10] Schmitz R, Wright GW, Huang DW, Johnson CA, Phelan JD, Wang JQ (2018). Genetics and Pathogenesis of Diffuse Large B-Cell Lymphoma. N Engl J Med.

[11] Cho SF, Yeh TJ, Wang HC, Du JS, Gau YC, Lin YY (2024). Prognostic mutation signature would serve as a potential prognostic predictor in patients with diffuse large B-cell lymphoma. Sci Rep.

[12] Eertink JJ, Zwezerijnen GJC, Heymans MW, Pieplenbosch S, Wiegers SE, Duhrsen U (2023). Baseline PET radiomics outperforms the IPI risk score for prediction of outcome in diffuse large B-cell lymphoma. Blood.

[13] Bradshaw PT (2024). Body composition and cancer survival: a narrative review. Br J Cancer.

[14] Petrelli F, Cortellini A, Indini A, Tomasello G, Ghidini M, Nigro O (2021). Association of Obesity With Survival Outcomes in Patients With Cancer: A Systematic Review and Meta-analysis. JAMA Netw Open.

[15] Wang J, Tan S, Gianotti L, Wu G (2023). Evaluation and management of body composition changes in cancer patients. Nutrition.

[16] Guo Y, Luan H, Lin J (2026). Obesity is the culprit behind fatty acid-induced inflammation. Nutr Metab (Lond).

[17] Sun Y, Lin X, Zou Z, Zhou Y, Liu A, Li X (2025). Association between visceral fat area and metabolic syndrome in individuals with normal body weight: insights from a Chinese health screening dataset. Lipids Health Dis.

[18] Charles-Messance H, Mitchelson KAJ, De Marco Castro E, Sheedy FJ, Roche HM (2020). Regulating metabolic inflammation by nutritional modulation. J Allergy Clin Immunol.

[19] Cozzo AJ, Fuller AM, Makowski L (2017). Contribution of Adipose Tissue to Development of Cancer. Compr Physiol.

[20] Quail DF, Dannenberg AJ (2019). The obese adipose tissue microenvironment in cancer development and progression. Nat Rev Endocrinol.

[21] Bouche C, Quail DF (2023). Fueling the Tumor Microenvironment with Cancer-Associated Adipocytes. Cancer Res.

[22] Bacci M, Lorito N, Smiriglia A, Morandi A (2021). Fat and Furious: Lipid Metabolism in Antitumoral Therapy Response and Resistance. Trends Cancer.

[23] Fleming CA, O'Connell EP, Kavanagh RG, O'Leary DP, Twomey M, Corrigan MA (2021). Body Composition, Inflammation, and 5-Year Outcomes in Colon Cancer. JAMA Netw Open.

[24] Peila R, Rohan TE (2025). MRI Measures of Fat Distribution and Risk of Cancer. Cancer Epidemiol Biomarkers Prev.

[25] Li L, Li W, Xu D, He H, Yang W, Guo H (2023). Association Between Visceral Fat Area and Cancer Prognosis: A Population-Based Multicenter Prospective Study. Am J Clin Nutr.

[26] Eide AJ, Halle MK, Lura N, Fasmer KE, Wagner-Larsen K, Forsse D (2023). Visceral fat percentage for prediction of outcome in uterine cervical cancer. Gynecol Oncol.

[27] Li WF, Que CR, Xu DB, Li P (2025). Impact of visceral fat distribution on postoperative complications in high-aged patients undergoing gastric cancer surgery: A cross-sectional study. World J Gastroenterol.

[28] Moon HG, Ju YT, Jeong CY, Jung EJ, Lee YJ, Hong SC (2008). Visceral obesity may affect oncologic outcome in patients with colorectal cancer. Ann Surg Oncol.

[29] Li Y, Yu Y, Lv K, Ge R, Xie X (2025). Prognostic value of body adipose tissue parameters in cancer patients treated with immune checkpoint inhibitors. Front Immunol.

[30] Poltronieri TS, Persico RS, Viana LV (2024). Body adipose tissue depots and treatment outcomes for women with breast cancer: A systematic review. Clin Nutr.

[31] Go SI, Park MJ, Song HN, Kim HG, Kang MH, Lee HR (2016). Prognostic impact of sarcopenia in patients with diffuse large B-cell lymphoma treated with rituximab plus cyclophosphamide, doxorubicin, vincristine, and prednisone. J Cachexia Sarcopenia Muscle.

[32] Besutti G, Massaro F, Bonelli E, Braglia L, Casali M, Versari A (2021). Prognostic Impact of Muscle Quantity and Quality and Fat Distribution in Diffuse Large B-Cell Lymphoma Patients. Front Nutr.

[33] Shin DY, Kim A, Byun BH, Moon H, Kim S, Ko YJ (2016). Visceral adipose tissue is prognostic for survival of diffuse large B cell lymphoma treated with frontline R-CHOP. Ann Hematol.

[34] Chen Y, Chen Z, Tan X, Zhang Q, Zhou Y, Yuan H (2023). Role of body composition and metabolic parameters extracted from baseline (18)F-FDG PET/CT in patients with diffuse large B-cell lymphoma. Ann Hematol.

[35] Kapoor ND, Twining PK, Groot OQ, Pielkenrood BJ, Bongers MER, Newman ET (2020). Adipose tissue density on CT as a prognostic factor in patients with cancer: a systematic review. Acta Oncol.

[36] Sottier D, Petit JM, Guiu S, Hamza S, Benhamiche H, Hillon P (2013). Quantification of the visceral and subcutaneous fat by computed tomography: interobserver correlation of a single slice technique. Diagn Interv Imaging.

[37] Himbert C, Ose J, Nattenmüller J, Warby CA, Holowatyj AN, Böhm J (2019). Body Fatness, Adipose Tissue Compartments, and Biomarkers of Inflammation and Angiogenesis in Colorectal Cancer: The ColoCare Study. Cancer Epidemiol Biomarkers Prev.

[38] Geng JH, Tu HP, Shih PM, Shen JT, Jang MY, Wu WJ (2015). Noncontrast computed tomography can predict the outcome of shockwave lithotripsy via accurate stone measurement and abdominal fat distribution determination. Kaohsiung J Med Sci.

[39] Chen J, Mei J, Li X, Lu Y, Yu Q, Wei Q (2024). TransUNet: Rethinking the U-Net architecture design for medical image segmentation through the lens of transformers. Med Image Anal.

[40] Chang C-H (2024). Optimizing Learning Efficiency in Abdominal Computed Tomography Image Segmentation with the EfficientTransUNet Model [Master's thesis]. Kaohsiung, Taiwan: National Sun Yat-sen University.

[41] Sparreboom A, Wolff AC, Mathijssen RH, Chatelut E, Rowinsky EK, Verweij J (2007). Evaluation of alternate size descriptors for dose calculation of anticancer drugs in the obese. J Clin Oncol.

[42] Cespedes Feliciano EM, Chen WY, Lee V, Albers KB, Prado CM, Alexeeff S (2020). Body Composition, Adherence to Anthracycline and Taxane-Based Chemotherapy, and Survival After Nonmetastatic Breast Cancer. JAMA Oncol.

[43] Li Z, Ngu R, Naik AA, Trinh K, Paharkova V, Liao H (2024). Adipocyte maturation impacts daunorubicin disposition and metabolism. Eur J Clin Invest.

[44] Santoro A, Kahn BB (2023). Adipocyte Regulation of Insulin Sensitivity and the Risk of Type 2 Diabetes. N Engl J Med.

[45] Shen S, Brown KA, Green AK, Iyengar NM (2026). Obesity and Cancer: A Translational Science Review. JAMA.

[46] Uddin S, Bu R, Ahmed M, Hussain AR, Ajarim D, Al-Dayel F (2010). Leptin receptor expression and its association with PI3K/AKT signaling pathway in diffuse large B-cell lymphoma. Leuk Lymphoma.

[47] Lam QL, Wang S, Ko OK, Kincade PW, Lu L (2010). Leptin signaling maintains B-cell homeostasis via induction of Bcl-2 and Cyclin D1. Proc Natl Acad Sci U S A.

[48] Sheng X, Parmentier JH, Tucci J, Pei H, Cortez-Toledo O, Dieli-Conwright CM (2017). Adipocytes Sequester and Metabolize the Chemotherapeutic Daunorubicin. Mol Cancer Res.

[49] Hohloch K, Altmann B, Pfreundschuh M, Loeffler M, Schmitz N, Zettl F (2018). Obesity negatively impacts outcome in elderly female patients with aggressive B-cell lymphomas treated with R-CHOP: results from prospective trials of the German high grade non-Hodgkin's lymphoma trial group. Br J Haematol.

[50] Muller C, Murawski N, Wiesen MH, Held G, Poeschel V, Zeynalova S (2012). The role of sex and weight on rituximab clearance and serum elimination half-life in elderly patients with DLBCL. Blood.

[51] Ternant D, Monjanel H, Venel Y, Prunier-Aesch C, Arbion F, Colombat P (2019). Nonlinear pharmacokinetics of rituximab in non-Hodgkin lymphomas: A pilot study. Br J Clin Pharmacol.

[52] Sawalha Y, Rouphail B, Jia X, Dean RM, Hill BT, Jagadeesh D (2016). Is rituximab sub-optimally dosed in indolent B cell lymphoma?. Br J Haematol.

[53] Sehn LH, Berry B, Chhanabhai M, Fitzgerald C, Gill K, Hoskins P (2007). The revised International Prognostic Index (R-IPI) is a better predictor of outcome than the standard IPI for patients with diffuse large B-cell lymphoma treated with R-CHOP. Blood.

[54] Wieringa A, Boslooper K, Hoogendoorn M, Joosten P, Beerden T, Storm H (2014). Comorbidity is an independent prognostic factor in patients with advanced-stage diffuse large B-cell lymphoma treated with R-CHOP: a population-based cohort study. Br J Haematol.

[55] Steyerberg EW, Pencina MJ, Lingsma HF, Kattan MW, Vickers AJ, Van Calster B (2012). Assessing the incremental value of diagnostic and prognostic markers: a review and illustration. Eur J Clin Invest.

[56] Moons KG, Altman DG, Reitsma JB, Ioannidis JP, Macaskill P, Steyerberg EW (2015). Transparent Reporting of a multivariable prediction model for Individual Prognosis or Diagnosis (TRIPOD): explanation and elaboration. Ann Intern Med.

