# Supplementary material for: Associations of Subcutaneous and Visceral Fat Areas with Progression-Free Survival in Patients with Diffuse Large B-Cell Lymphoma
Source: Int J Med Sci. 2026 Sep 24;23(11):3415–23. doi: 10.7150/ijms.137961 (PMC13685037; doi:10.7150/ijms.137961)
Supplement: Supplementary file 1 [file ijmsv23p3415s1.pdf]

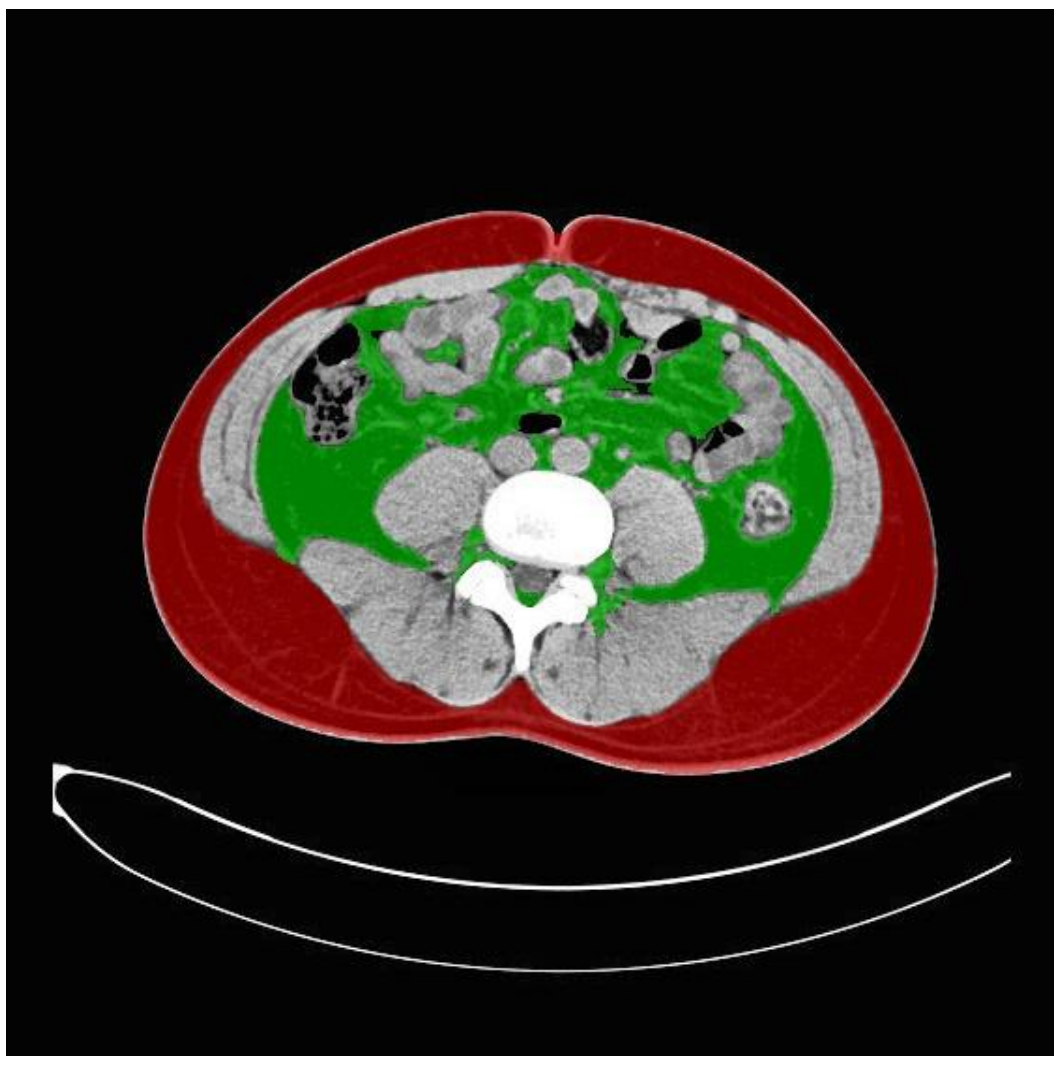

(A)

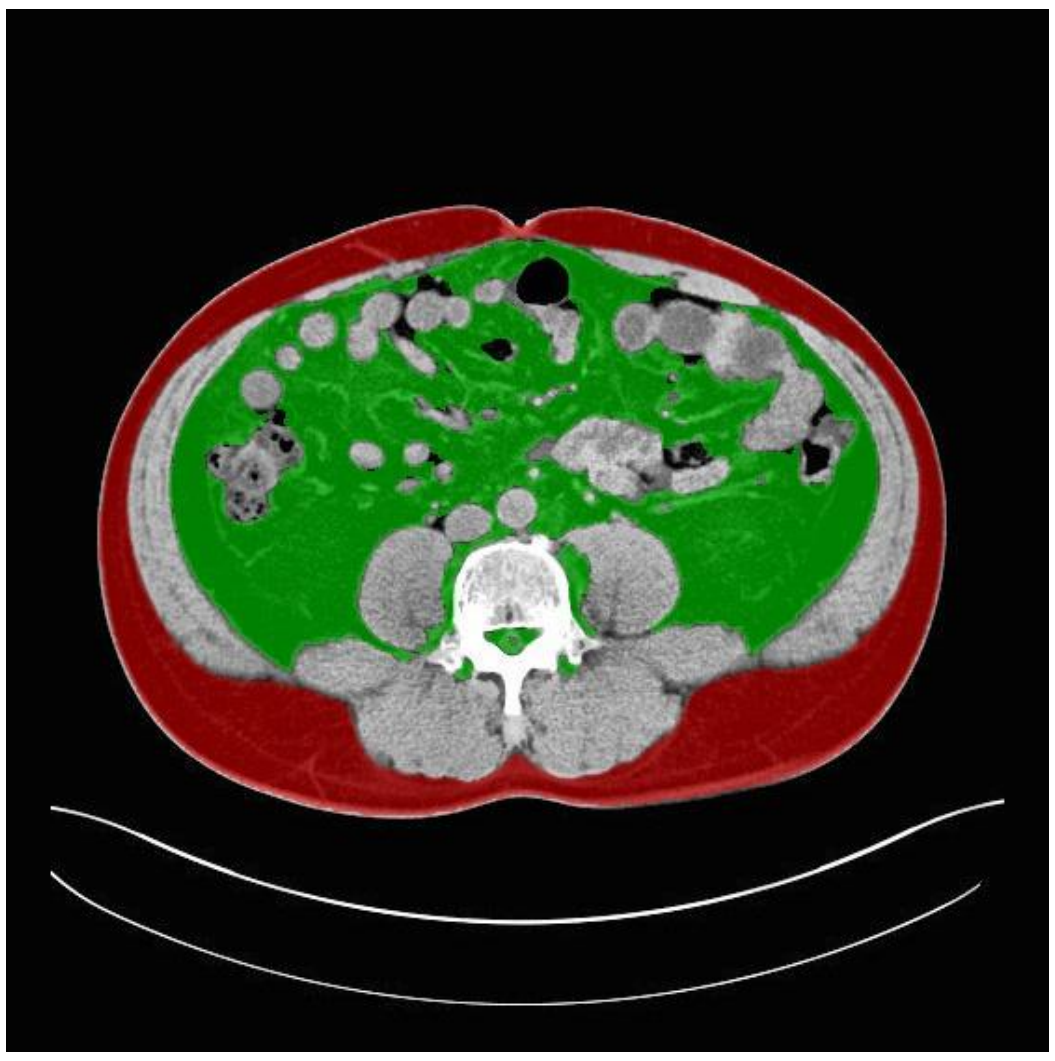

(B)

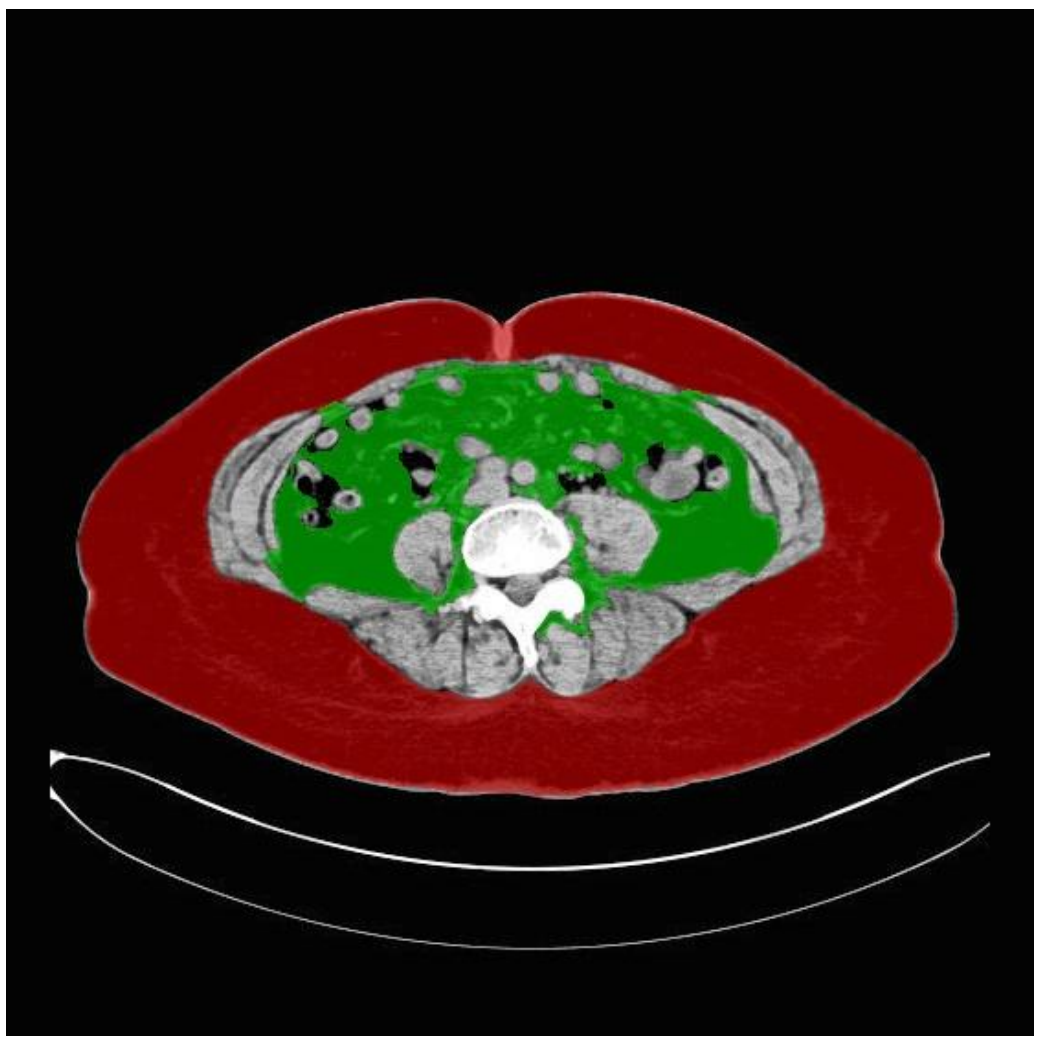

(C)

Supplementary Figure S1. Representative Computed Tomography Images of Automated Visceral and Subcutaneous Adipose Tissue Segmentation. Representative axial computed tomography images demonstrate (A) a relatively balanced distribution of visceral and subcutaneous adipose tissue, (B) a visceral fat–dominant distribution, and (C) a subcutaneous fat–dominant distribution. Visceral and subcutaneous adipose tissue are displayed in green and red, respectively. Abbreviations: VFA, visceral fat area; SFA, subcutaneous fat area.

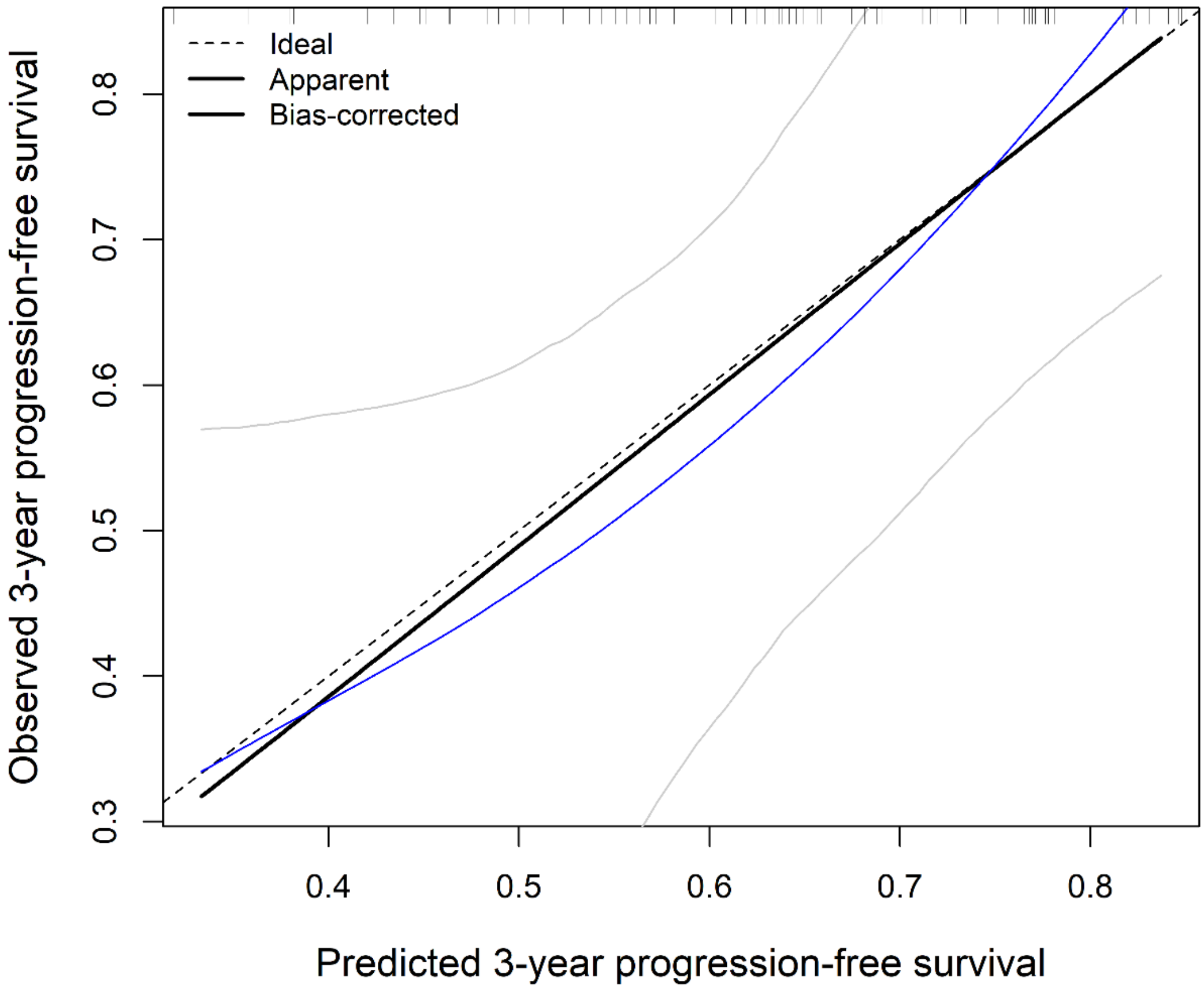

Supplementary Figure S2. Bootstrap-Corrected Calibration of the IPI-Plus-SFA Model for 3-Year Progression-Free Survival. Calibration of the IPI-plus-SFA Cox proportional hazards model for 3-year progression-free survival was assessed using 1,000 bootstrap resamples. The apparent and optimism-corrected calibration curves are shown in relation to the ideal line of perfect agreement between predicted and observed probabilities. Abbreviations: PFS, progression-free survival; IPI, International Prognostic Index; SFA, subcutaneous fat area.

Supplementary Table S1. Sensitivity Analyses of the Association Between Subcutaneous Fat Area and Progression-Free Survival

Model	Adjustment variables	HR per 1-SD increase in SFA	95% CI	*p* value
Primary model	IPI	1.34	1.00–1.79	0.047
Clinical sensitivity model	IPI, CCI, treatment regimen	1.52	1.11–2.08	0.009
Body-size and adiposity sensitivity model	IPI, VFA, BMI	1.22	0.87–1.70	0.250

Abbreviations: HR, hazard ratio; CI, confidence interval; SD, standard deviation; SFA, subcutaneous fat area; VFA, visceral fat area; BMI, body mass index; IPI, International Prognostic Index; CCI, Charlson Comorbidity Index.

Note: SFA was analyzed as a continuous variable standardized to a 1-SD increment. The primary model was adjusted for IPI. The clinical sensitivity model additionally included CCI and treatment regimen, and the body-size and adiposity sensitivity model additionally included VFA and BMI.

Supplementary Table S2. Exploratory Associations of Subcutaneous and Visceral Fat Areas With Overall Survival

Model	Variable	HR per 1-SD increase	95% CI	p value
Separate IPI-adjusted models	SFA	1.13	0.82–1.54	0.459
	VFA	1.07	0.79–1.43	0.678
Joint fat-compartment model	SFA	1.12	0.81–1.54	0.507
	VFA	1.04	0.76–1.42	0.799

Abbreviations: HR, hazard ratio; CI, confidence interval; SD, standard deviation; SFA, subcutaneous fat area; VFA, visceral fat area; IPI, International Prognostic Index.

Note: Analyses included 109 patients with complete data, among whom 43 deaths occurred. SFA and VFA were standardized and modeled as continuous variables. Separate models included the IPI and either SFA or VFA, whereas the joint model included the IPI, SFA, and VFA simultaneously. The standardized SFA and VFA coefficients in the joint model did not differ significantly (Wald test, $p = 0.784$).
